# Thymic hyperplasia after autologous hematopoietic stem cell transplantation in multiple sclerosis: a case series

**DOI:** 10.3389/fimmu.2024.1478777

**Published:** 2024-11-25

**Authors:** Alice Mariottini, Riccardo Boncompagni, Diletta Cozzi, Edoardo Simonetti, Anna Maria Repice, Valentina Damato, Mirella Giordano, Vittorio Miele, Chiara Nozzoli, Luca Massacesi

**Affiliations:** ^1^ Department of Neurosciences, Psychology, Drug Research and Child Health (NEUROFARBA), University of Florence, Florence, Italy; ^2^ Neurology II Department, University Hospital Careggi, Florence, Italy; ^3^ Cell Therapy and Transfusion Medicine Unit, University Hospital Careggi, Florence, Italy; ^4^ Department of Emergency Radiology, University Hospital Careggi, Florence, Italy

**Keywords:** multiple sclerosis, hematopoietic stem cell transplantation, transplant, thymus, immune reconstitution, autoimmune diseases, CT, imaging

## Abstract

**Introduction:**

Reactivation of thymopoiesis in adult patients with autoimmune disorders treated with autologous haematopoietic stem cell transplantation (AHSCT) is supported by studies exploring immunoreconstitution. Radiological evidence of thymic hyperplasia after AHSCT was previously reported in patients with systemic sclerosis, but, to our knowledge, it has not been described in multiple sclerosis (MS), where premature thymic involution has been observed and immunosenescence might be accelerated by disease-modifying treatments (DMTs).

**Participants and methods:**

monocentric case series including MS patients who performed a chest CT scan for clinical purposes after having received AHSCT (BEAM/ATG regimen) for aggressive MS failing DMTs. Chest CT exams were reviewed by a thoracic radiologist: thymic hyperplasia was defined as a rounded mass in the thymic loggia with a density around 40 Hounsfield Units (HU) and thickness >1.3 cm.

**Results:**

Fifteen MS patients were included; the median time interval between AHSCT and chest CT scan was 2 (range 1-18) months. All the patients were free from new inflammatory events and DMTs over a median follow-up of 36 months (range 12-84) after AHSCT. Thymic hyperplasia was detected in 3/15 (20%) cases in an exam taken 1 to 3 months after AHSCT; all these patients were females, and aged 30 to 40 years. Lung infections and secondary autoimmunity were diagnosed in 5 and 1 cases, respectively, none of which showed thymic hyperplasia. No associations between thymic hyperplasia and clinical-demographic characteristics or post-AHSCT outcomes were observed.

**Conclusions:**

Thymic hyperplasia was detected in 20% of MS patients recently treated with AHSCT. These results are consistent with previous immunological studies showing that AHSCT promotes thymus reactivation in MS patients, further supporting *de-novo* thymopoiesis as a cornerstone of immune reconstitution after AHSCT in this population.

## 1 Introduction

**Figure 1:**
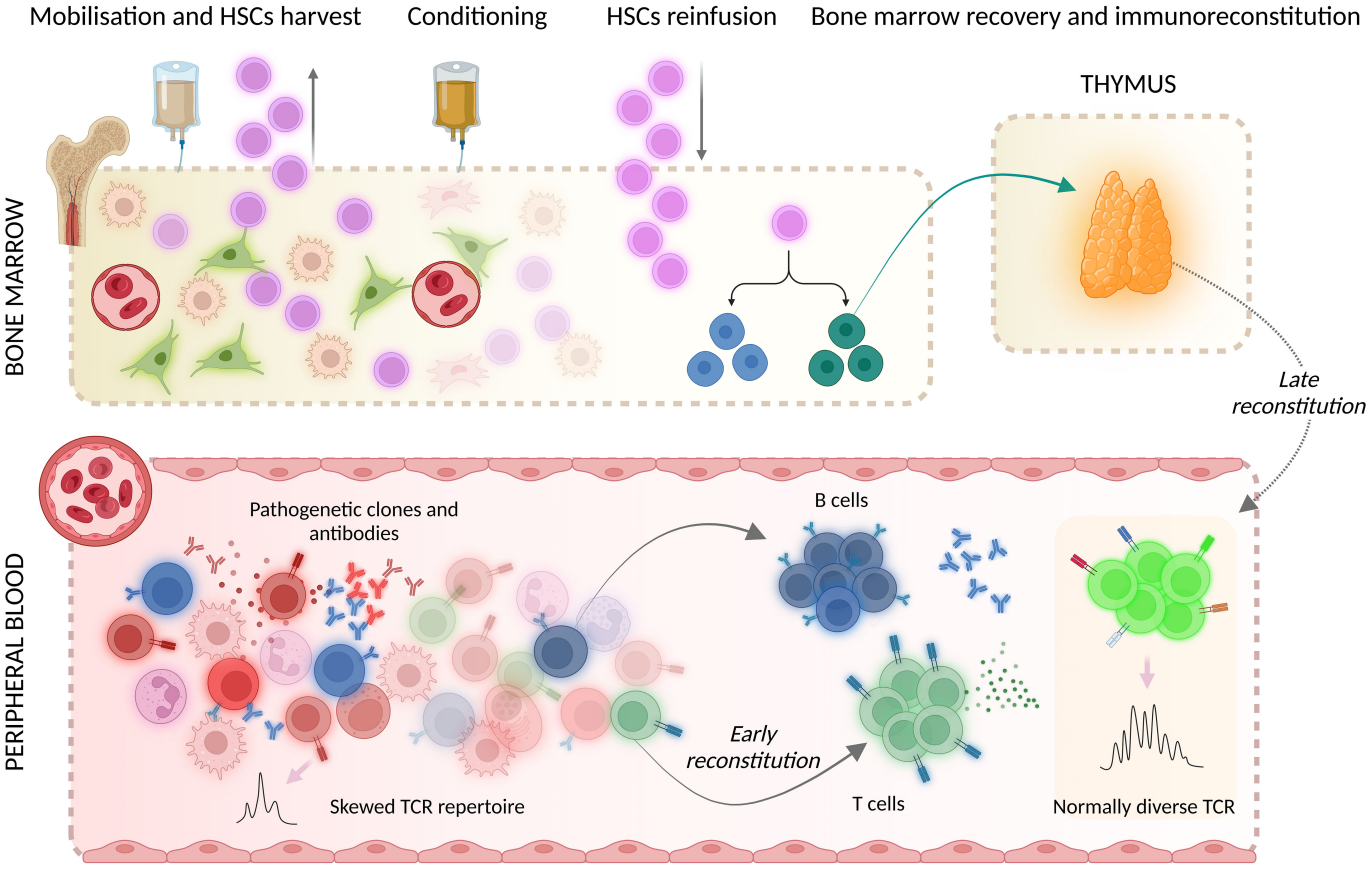
Mechanisms of immunoreconstitution after AHSCT encompass early reconstitution, promoted by the homeostatic expansion of cells that survived the conditioning chemotherapy, and late reconstitution, mainly prompted by *de-novo* thymopoiesis. HSCs, hematopoietic stem cells; TCR, T cell receptor. Created with BioRender.com. Mariottini, A. (2024) BioRender.com/n84b118.

Autologous haematopoietic stem cell transplantation (AHSCT) is currently endorsed as a treatment option for selected aggressive autoimmune disorders, including multiple sclerosis (MS), a chronic autoimmune demyelinating and neurodegenerative disease of the central nervous system (CNS) (1–3). AHSCT is a multistep procedure consisting of the administration of high-dose chemotherapy/serotherapy inducing the ablation of the haematolymphoid system, which is followed by immune reconstitution prompted by the reinfusion of haematopoietic stem cells (HSCs) previously collected from the individual itself. Immunoreconstitution after AHSCT occurs in two phases: (i) early reconstitution, mainly promoted by a homeostatic expansion of cells surviving the chemotherapy, and (ii) late reconstitution, which is promoted by *de-novo* thymopoiesis (**Figure 1**). The latter is associated with a radical renewal of the T cell compartment and therefore likely contributes to the efficacy of AHSCT in autoimmune disorders (4).

Despite physiological age-related thymic involution, acute shrinking of thymic tissue followed by growth to a volume similar to or even larger than baseline is seen after acute stress events such as infections, radiation therapy, surgery and burns (5). The most frequent cause of thymic rebound hyperplasia is chemotherapy, with 10-25% incidence usually within 2 years of treatment (6). Thymic rebound hyperplasia is more frequent in children and young adults (with a strictly age-related incidence) and may be challenging during radiological restaging of lymphoma patients with mediastinal involvement (7). Immunophenotyping of T cell populations demonstrated that this radiological finding is associated with an increase or resumption of thymus activity, representing the morphological counterpart of reactivation of thymopoiesis (8).

Radiological evidence of thymic hyperplasia in people undergoing AHSCT for autoimmune indication was previously reported in a cohort of patients with systemic sclerosis (9), but, to our knowledge, it has never been described in people with MS. As reduced thymic output suggestive of premature thymic involution was described in MS (10), and as this phenomenon may be accelerated by disease-modifying treatments (DMTs) (11), thymus response to the chemotherapy administered during AHSCT may differ in people with MS compared to patients affected by other autoimmune disorders.

The occurrence of thymic hyperplasia as detected by chest CT was therefore explored in a monocentric case series including MS patients previously treated with AHSCT after failure of conventional DMTs, who performed a chest CT scan for clinical purposes.

## 2 Participants and methods

### 2.1 Participants and AHSCT procedure

Patients affected by relapsing-remitting (RR-) or secondary-progressive (SP-) MS diagnosed according to the McDonald criteria (12–14) received AHSCT at the Cell Therapy and Transfusion Medicine Unit of the Careggi University Hospital in Florence, Italy, in collaboration with the MS Referral Centre for the Tuscany region of the same hospital. All the patients were treated with the same AHSCT protocol after being selected according to the inclusion/exclusion criteria of the centre, as previously reported (15). Briefly, Peripheral Blood Haematopoietic Stem Cells (PBSC) were mobilised by the administration of high doses of cyclophosphamide (4 g/sqm) followed by daily granulocyte colony-stimulating factor (G-CSF; 10 μg/kg per day) starting at day +5, until completion of the PBSC harvest by leukapheresis. Conditioning was performed with the myeloablative intermediate intensity regimen BEAM/ATG (3), which encompasses the following: BCNU (Carmustine) 300 mg/m^2^ on day −6, ARA-C (Cytosine-Arabinoside) 200 mg/m^2^/day and VP-16 (Etoposide) 200 mg/m^2^/day from day −5 to day −2, and Melphalan 140 mg/m^2^ on day −1; rabbit anti**-**thymocyte globulin (ATG, Thymoglobulin™, Sanofi) was added at a dose of 3.75 mg/kg/day on day +1 and +2 (total dose 7.5 mg/Kg). Supportive therapies and infection prophylaxis were administered according to local protocols.

### 2.2 Post-AHSCT follow-up

Standardised haematological and neurological evaluations were performed at baseline, at months 6 and 12 after AHSCT and then at least yearly. Immune cell reconstitution was determined by flow cytometry immunophenotyping performed in peripheral blood according to clinical practice, including a pre-AHSCT sample and longitudinal post-AHSCT follow-up. New focal inflammatory activity after AHSCT was defined as the occurrence of relapses and/or brain magnetic resonance imaging (MRI) activity (i.e. appearance of new T2 lesions at follow-up compared to the re-baseline scan taken at month six after AHSCT, or of gadolinium-enhancing lesions at any time).

### 2.3 Chest CT scan

Chest CT exams were performed with a new generation computed tomography (Revolution HD, General Electrics, 128 slice row) with the same protocol: a baseline scan followed by a chest acquisition after intravenous contrast media injection (acquisition in a venous phase, after 60 s from the injection). The axial acquisitions were reconstructed also in coronal and sagittal planes to better identify mediastinal structures. Chest CT exams were reviewed by a thoracic radiologist, blind to clinical data. CT venous images were then analysed in a post-processing phase by a dedicated software (SingoVia, Siemens – Erlangen, Germany), using specific panel visualization that help radiologists in applying morpho-volumetric analysis. Thymic hyperplasia was defined as a rounded mass in the thymic loggia with a density around 40 Hounsfield Units (HU) and thickness >1.3 cm (16, 17). Thymic rebound was defined as increased thickness not exceeding 1.3 cm and soft-tissue lobulation in the thymic loggia. Specific measurements of thymic gland were performed using two major perpendicular measures in the axial plane; where possible, the software allows a volumetric reconstruction.

### 2.4 Statistical methods

Continuous and dichotomous variables are summarised as median (range) or number (frequency), respectively. Correlations between thymic hyperplasia and clinical-demographic characteristics and post-AHSCT variables were explored using Spearman correlation analysis, considering as significant a two-tailed p-value <0.05. The statistical software used was SPSS version 25 for Windows.

## 3 Case presentation

### 3.1 Patient population

Fifteen MS patients (13 females; 7 RR-MS) were included (**Table 1**). All the patients had received DMTs before AHSCT, including interferons (n=12), natalizumab and fingolimod (n=8 each), anti-CD20 monoclonal antibodies and cyclophosphamide (n=6 each), glatiramer-acetate (n=4), dimethyl-fumarate (n=3), azathioprine, alemtuzumab and cladribine (n=1 each).

**Table 1:** Clinical-demographic characteristics of the MS patients included (n=15).

	median	(range)
Age at CT scan, years	40	(26 – 50)
MS duration at CT scan, years	16	(7 – 25)
Number of DMTs prior to AHSCT	3	(2 – 6)
Duration of treatment with DMTs before AHSCT, years	10	(2 – 21)
EDSS at AHSCT	5.0	(1.5 – 6.5)
Time interval between AHSCT and chest CT, months	2	(1 – 18)
	n	(%)
Sex, female	13	(87%)
MS form, relapsing-remitting	7	(47%)

AHSCT, autologous haematopoietic stem cell transplantation; CT, computed tomography; DMTs, disease-modifying treatments; EDSS, Expanded Disability Status Scale; MS, multiple sclerosis.

Chest CT scan was performed a median of 2 (1 – 18) months after AHSCT. The indication for CT exams was suspicion of lung infections in 12 (80%) cases; 2 (13%) patients performed total body CT scans for EBV-related post-transplant lymphoproliferative disorder (PTLD) stadiation, while 1 (7%) performed pulmonary CT angiography for monitoring of an intracardiac thrombosis that occurred before AHSCT. Chest CT scan was consistent with pneumonia in 6 out of 15 (40%) cases, and 5 patients performed a consecutive bronchoscopy with bronchoalveolar lavage, resulting in aetiological agent identification in 3 cases (1 case of Pneumocystis jirovecii, 1 Cytomegalovirus, 1 H1N1). A pre-AHSCT scan was available in 3 cases for AHSCT eligibility screening.

### 3.2 Thymus appearance on chest CT

**Figure 2:**
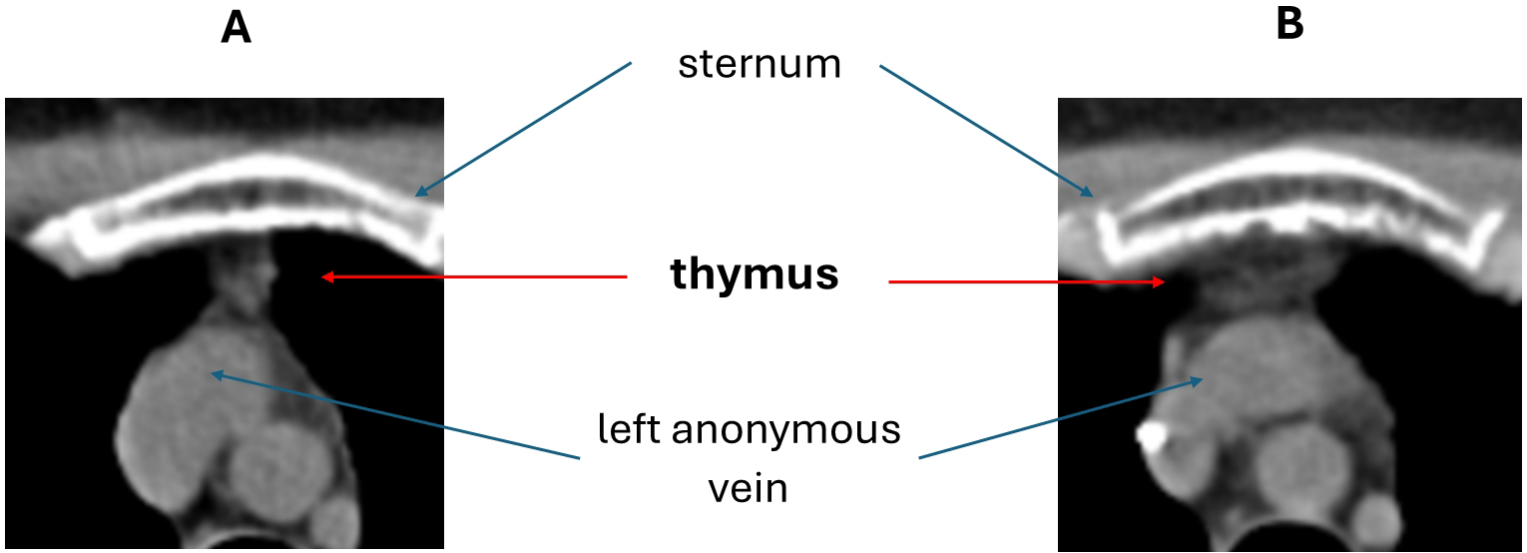
Example of thymic hyperplasia detected by chest CT scan after AHSCT **(B)** compared to a pre-AHSCT exam **(A)**.

Thymic hyperplasia (**Figure 2**) was detected in 3/15 (20%) patients in CT exams taken 1 to 3 months after AHSCT. The time interval between AHSCT and CT was 1 month (range 1 – 3) in patients with thymic hyperplasia and 3 months (range 1 – 18) in those without. All the patients showing thymic hyperplasia were females and affected by SP-MS; they were aged 30 to 40 years. Previous DMTs received by these three patients included interferons, natalizumab, and cyclophosphamide (n=2 each); glatiramer-acetate, dimethyl-fumarate, alemtuzumab, and fingolimod (n=1 each). Follow-up imaging was available in one patient, who showed a further increase in thymus size six months later (month 7 after AHSCT) followed by stabilisation at 1 year after AHSCT.

Four further patients (27%) showed a thymic rebound in a CT exam taken a median of 11 months (range 1 – 18) after AHSCT. Three out of 4 patients were females (75%), and two were affected by RR-MS; the median age at CT scan was 47.5 years (range 28 – 50).

### 3.3 T cell reconstitution and outcomes after AHSCT

**Figure 3:**
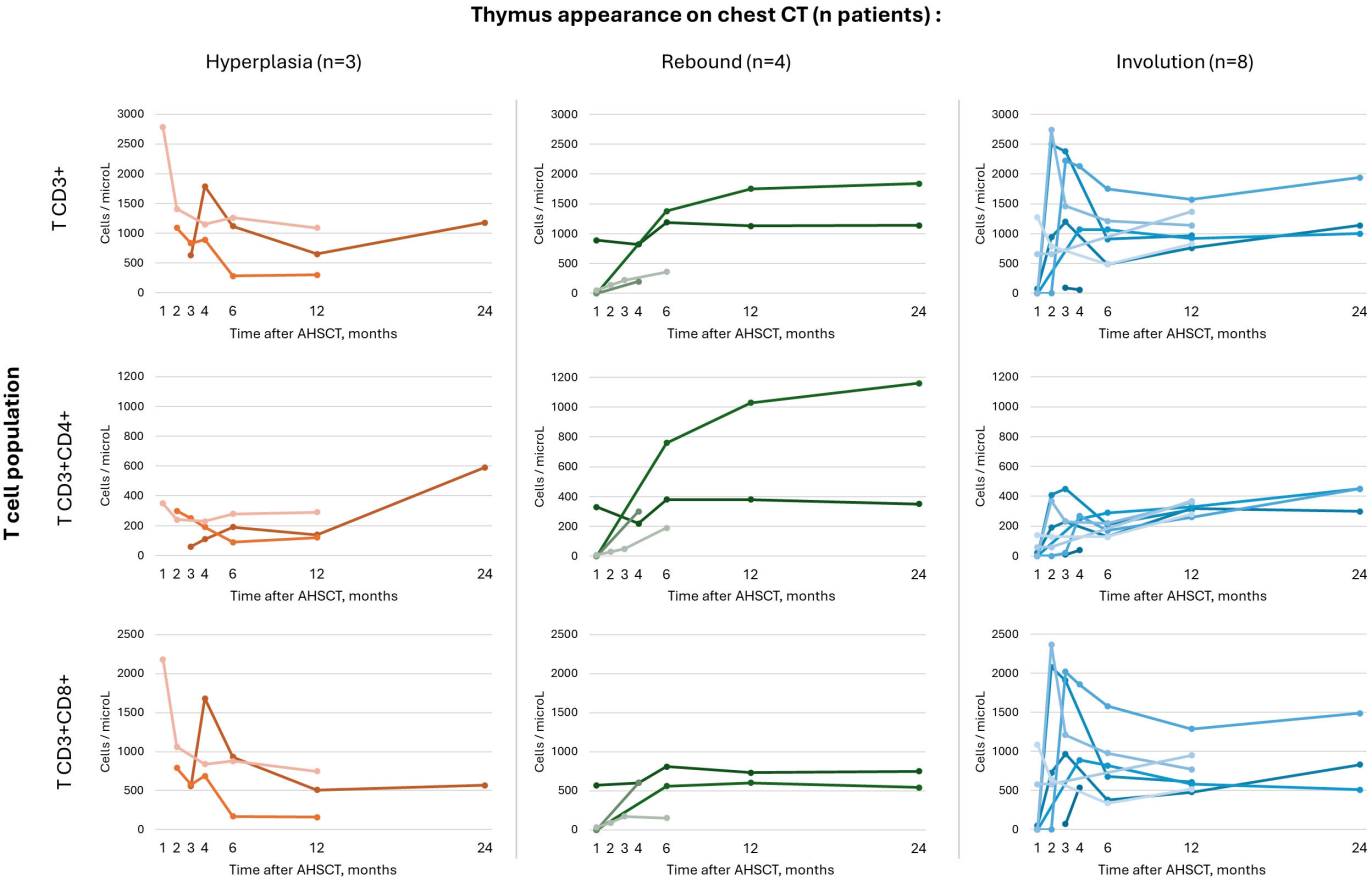
Kinetics of repopulation of CD3+ lymphocyte and CD4+ and CD8+ subsets after AHSCT in individual patients stratified according to thymus appearance on chest CT scan, i.e. hyperplasia (n=3), rebound (n=4) or involution (n=8).

Kinetics of reconstitution of CD3+ lymphocyte subsets after AHSCT are shown in **Figure 3**. Individual trajectories of patients with thymic hyperplasia/rebound suggest a trend towards a later repopulation of T cells compared to those with thymus involution, who show an early increase, especially in CD8+ T cell count; no statistical analysis was performed due to the small sample size and missing timepoints for some patients.

All the patients were free from new focal inflammatory events (relapses or new T2 and/or gadolinium enhancing MRI lesions) and from treatment with DMTs over a median follow-up of 36 months (range 12 - 84) after AHSCT. At the latest follow-up, EDSS worsened in 8/15 cases, one of which showed thymic hyperplasia. Secondary autoimmunity was diagnosed in one patient (haemolytic anaemia), who showed thymic rebound. EBV reactivations requiring treatment were observed in 5/15 patients (33%), being thymic hyperplasia observed in 1/5 cases. None of the patients with thymic hyperplasia was diagnosed with lung infection.

No associations between thymic hyperplasia and clinical-demographic characteristics or post-AHSCT outcomes were observed.

## 4 Discussion

Age-related thymic atrophy/involution is a well-known physiologic process starting in the first year of life (18), and the vast majority of adults show complete fatty replacement of the thymus on chest CT scans (19). However, thymic hyperplasia may be observed in adults after exposure to definite triggers, including COVID-19 (20), and the chemotherapy administered for the treatment of malignancies and during AHSCT (21, 22). Intriguingly, in the context of COVID-19 infection, thymus enlargement was associated with increased T lymphocyte production estimated by sj/βTREC ratio, which correlated with the CT scan thymic score; this was interpreted by the Authors as a beneficial adaptation of the thymus to virus-induced lymphopenia (23). In one study including patients with mature B cell lymphoma treated with chemotherapy, patients with thymic hyperplasia showed a faster recovery of sjTREC levels and CD31+ recent thymic emigrants (RTE) counts than patients with comparable age, gender, diagnosis, disease stage, thymic volume and output function at baseline but without thymic hyperplasia; furthermore, they had a faster repopulation of both naïve CD4+ T cell and natural regulatory CD4+ T cell subsets than those without thymic hyperplasia (8). Such observations overall suggest that thymic hyperplasia reported in these different conditions could be associated with a renewal of thymopoiesis. In the autoimmune setting, an increase in thymic size from baseline associated with an increase in recent thymic emigrants (TREC+ T cells) was first detected between 3 and 12 months after AHSCT in patients with systemic sclerosis (9). Thymic hypertrophy showed an age-dependent pattern, as it was significantly greater at 1 and 2 years post-AHSCT in patients aged less than 43 years than in older patients; nonetheless, TREC+ T cells were increased also in the latter population, although at a lower extent (9).

In the present case series, thymic hyperplasia was detected in 3/15 MS patients, and 4 further cases showed thymic rebound. All the patients with thymic hyperplasia were younger than 41 years, but 3 out of four patients with thymic rebound were older than 45 years. No associations between thymic hyperplasia and secondary autoimmunity were detected, although the number of events observed was small, and associations with response to AHSCT could not be explored as no new focal inflammatory events were observed after transplant. The radiological evidence of thymic hyperplasia in this population is somehow expected based on previous observation of increased frequency of naïve T cells and recent thymic emigrants in MS treated with AHSCT, as well as of T cell receptor (TCR) repertoire renewal compared to pre-treatment, all supporting *de-novo* thymopoiesis (24, 25). The timing of thymic hyperplasia observed in our patients is likely consistent with known dynamics of T cell maturation within the thymus, considering that (i) about 3 weeks are required between the entry of a T-cell progenitor into the thymus and the export of its mature progeny in mice (26), and (ii) TRECs+ T cell counts in humans increase starting from month 3 after AHSCT (9).

Nonetheless, evidence of thymic hyperplasia in 20% of patients from this case series is relevant as it suggests that people with MS can experience thymus reactivation at a rate similar to people undergoing chemotherapy for malignancy (6), despite bearing a prematurely involved thymus (10). This latter phenomenon is supported by evidence of reduced rates of production of recent thymic emigrants (27–30) and increased restriction of the TCR repertoire in MS patients compared to the healthy population (31). Interestingly, premature thymic involution was speculated to play a role in MS pathogenesis, as the development of experimental autoimmune encephalomyelitis correlated with progressive ultrastructural alterations within the thymus, characterised by progressive degeneration of both epithelial cells and thymocytes (32). Premature thymic involution could promote autoimmunity through an unbalance between thymopoiesis and homeostatic proliferation favouring the latter, with subsequent increase in the frequency of potentially autoreactive T cells; furthermore, accelerated cellular senescence was speculated to contribute to MS progression (10, 33).

In MS patients, thymic senescence may be further accelerated by treatment with DMTs, some of which (including alemtuzumab and fingolimod) have been demonstrated to reduce thymic output compared to pre-treatment baseline (11), an event that likely contributes to increased risk for infective complications, especially in aged MS patients (34). If AHSCT can effectively restore thymopoiesis in a patient population previously exposed to several DMTs, like the one described in this paper, it could also revert patients’ vulnerability to such complications. As a consequence, restoration of DMTs after AHSCT, when needed, might be safer than expected in this regard.

The main limitation of this case series is the lack of data on recent thymic emigrants and TCR repertoire, preventing us from unequivocally interpreting thymic hyperplasia as an imaging correlate of immune reconstitution, as it could be triggered by different events, including COVID-19 and chemotherapy. Nonetheless, previous studies showed that thymic hyperplasia was associated with increased thymic function in these conditions (8, 23), and the association of thymic hyperplasia as detected by chest CT with immunological markers of recent thymic maturation has previously been described in the autoimmune setting (9). In addition, the long-term response to AHSCT (in terms of absence of new focal inflammatory) activity observed in our patients suggests that effective renovation of the immune system took place in all the cases. Other limitations include a potential selection bias, as indication for CT scan was mostly suspicion of lung infection and PTLD; however, no associations were observed between thymic hyperplasia and confirmed lung infections or EBV reactivations, although this could be due to the small sample size. Lastly, the heterogeneous timing of CT scans and lack of systematic longitudinal follow-up might have prevented us from detecting thymic hyperplasia in some cases.

In conclusion, the present case series provides evidence of thymic hyperplasia in 20% of MS patients treated with AHSCT, further supporting the role of *de-novo* thymopoiesis as a cornerstone of the mechanism of action of AHSCT in autoimmune disorders.

## Data Availability

The datasets presented in this article are not readily available because the original contributions presented in the study are included in the article/supplementary material, further inquiries can be directed to the corresponding author/s. Requests to access the aggregated data should be directed to alice.mariottini@unifi.it.

## References

[1] Thompson AJ Baranzini SE Geurts J Hemmer B Ciccarelli O. Multiple sclerosis. Lancet. (2018) 391:1622–36. doi: 10.1016/S0140-6736(18)30481-1 29576504

[2] Cohen JA Baldassari LE Atkins HL Bowen JD Bredeson C Carpenter PA. Autologous hematopoietic cell transplantation for treatment-refractory relapsing multiple sclerosis: position statement from the American society for blood and marrow transplantation. Biol Blood Marrow Transplant. (2019) 25:845–54. doi: 10.1016/j.bbmt.2019.02.014 30794930

[3] Sharrack B Saccardi R Alexander T Badoglio M Burman J Farge D. Autologous haematopoietic stem cell transplantation and other cellular therapy in multiple sclerosis and immune-mediated neurological diseases: updated guidelines and recommendations from the EBMT Autoimmune Diseases Working Party (ADWP) and the Joint Accreditation Committee of EBMT and ISCT (JACIE). Bone Marrow Transplant. (2020) 55(2):283–306. doi: 10.1038/s41409-019-0684-0 31558790 PMC6995781

[4] Arruda LC Clave E Moins-Teisserenc H Douay C Farge D Toubert A. Resetting the immune response after autologous hematopoietic stem cell transplantation for autoimmune diseases. Curr Res Transl Med. (2016) 64:107–13. doi: 10.1016/j.retram.2016.03.004 27316394

[5] Webb WR Higgins CB. Thoracic imaging: pulmonary and cardiovascular radiology. Lippincott Williams & Wilkins (2011).

[6] Kissin CM Husband JE Nicholas D Eversman W. Benign thymic enlargement in adults after chemotherapy: CT demonstration. Radiology. (1987) 163:67–70. doi: 10.1148/radiology.163.1.3823458 3823458

[7] Franke FC Damek A Steglich J Kurch L Hasenclever D Georgi TW. Differentiation between rebound thymic hyperplasia and thymic relapse after chemotherapy in pediatric Hodgkin lymphoma. Pediatr Blood Cancer. (2023) 70:e30421. doi: 10.1002/pbc.30421 37243889

[8] Sun D-P Jin H Ding C-Y Liang J-H Wang L Fan L. Thymic hyperplasia after chemotherapy in adults with mature B cell lymphoma and its influence on thymic output and CD4+ T cells repopulation. Oncoimmunology. (2016) 5:e1137417. doi: 10.1080/2162402X.2015.1137417 27467956 PMC4910735

[9] Storek J Zhao Z Lin E Berger T McSweeney PA Nash RA. Recovery from and consequences of severe iatrogenic lymphopenia (induced to treat autoimmune diseases). Clin Immunol. (2004) 113:285–98. doi: 10.1016/j.clim.2004.07.006 PMC2956741 15507394

[10] Haegert D. Premature thymic involution and multiple sclerosis. J Neurol Neurophysiol. (2014) 5:2. doi: 10.4172/2155-9562.1000207

[11] Paghera S Sottini A Previcini V Capra R Imberti L. Age-related lymphocyte output during disease-modifying therapies for multiple sclerosis. Drugs Aging. (2020) 37:739–46. doi: 10.1007/s40266-020-00789-4 32761321

[12] Polman CH Reingold SC Edan G Filippi M Hartung HP Kappos L. Diagnostic criteria for multiple sclerosis: 2005 revisions to the "McDonald Criteria. Ann Neurol. (2005) 58:840–6. doi: 10.1002/ana.20703 16283615

[13] Polman CH Reingold SC Banwell B Clanet M Cohen JA Filippi M. Diagnostic criteria for multiple sclerosis: 2010 revisions to the McDonald criteria. Ann Neurol. (2011) 69:292–302. doi: 10.1002/ana.22366 21387374 PMC3084507

[14] Thompson AJ Banwell BL Barkhof F Carroll WM Coetzee T Comi G. Diagnosis of multiple sclerosis: 2017 revisions of the McDonald criteria. Lancet Neurol. (2018) 17:162–73. doi: 10.1016/S1474-4422(17)30470-2 29275977

[15] Mariottini A Marchi L Innocenti C Di Cristinzi M Pasca M Filippini S. Intermediate-intensity autologous hematopoietic stem cell transplantation reduces serum neurofilament light chains and brain atrophy in aggressive multiple sclerosis. Front Neurol. (2022) 13. doi: 10.3389/fneur.2022.820256 PMC8907141 35280289

[16] Nishino M Ashiku SK Kocher ON Thurer RL Boiselle PM Hatabu H. The thymus: a comprehensive review. Radiographics. (2006) 26:335–48. doi: 10.1148/rg.262045213 16549602

[17] Araki T Sholl LM Gerbaudo VH Hatabu H Nishino M. Imaging characteristics of pathologically proven thymic hyperplasia: identifying features that can differentiate true from lymphoid hyperplasia. Am J Roentgenology. (2014) 202:471–8. doi: 10.2214/AJR.13.11210 PMC4020512 24555583

[18] Gui J Mustachio LM Su D-M Craig RW. Thymus size and age-related thymic involution: early programming, sexual dimorphism, progenitors and stroma. Aging Dis. (2012) 3:280. 22724086 PMC3375084

[19] Araki T Nishino M Gao W Dupuis J Hunninghake GM Murakami T. Normal thymus in adults: appearance on CT and associations with age, sex, BMI and smoking. Eur Radiol. (2016) 26:15–24. doi: 10.1007/s00330-015-3796-y 25925358 PMC4847950

[20] Samir A Bastawi RA Baess AI Sweed RA Eldin OE. Thymus CT-grading and rebound hyperplasia during COVID-19 infection: a CT volumetric study with multivariate linear regression analysis. Egyptian J Radiol Nucl Med. (2022) 53:112. doi: 10.1186/s43055-022-00784-2

[21] Hakim FT Memon SA Cepeda R Jones EC Chow CK Kasten-Sportes C. Age-dependent incidence, time course, and consequences of thymic renewal in adults. J Clin Invest. (2005) 115:930–9. doi: 10.1172/JCI200522492 PMC1064981 15776111

[22] Yarom N Zissin R Apter S Hertz M Levene NR Gayer G. Rebound thymic enlargement on CT in adults. Int J Clin Pract. (2007) 61:562–8. doi: 10.1111/j.1742-1241.2006.00950.x 17263694

[23] Cuvelier P Roux H Couëdel-Courteille A Dutrieux J Naudin C Charmeteau de Muylder B. Protective reactive thymus hyperplasia in COVID-19 acute respiratory distress syndrome. Crit Care. (2021) 25:4. doi: 10.1186/s13054-020-03440-1 33397460 PMC7781174

[24] Muraro PA Douek DC Packer A Chung K Guenaga FJ Cassiani-Ingoni R. Thymic output generates a new and diverse TCR repertoire after autologous stem cell transplantation in multiple sclerosis patients. J Exp Med. (2005) 201:805–16. doi: 10.1084/jem.20041679 PMC2212822 15738052

[25] Harris KM Lim N Lindau P Robins H Griffith LM Nash RA. Extensive intrathecal T cell renewal following hematopoietic transplantation for multiple sclerosis. JCI Insight. (2020) 5(2):e127655. doi: 10.1172/jci.insight.127655 31877116 PMC7098711

[26] Janeway CA Jr Travers P Walport M Shlomchik MJ. Generation of lymphocytes in bone marrow and thymus., Immunobiology: The Immune System in Health and Disease. New York: Garland Science (2001).

[27] Duszczyszyn DA Williams JL Mason H Lapierre Y Antel J Haegert DG. Thymic involution and proliferative T-cell responses in multiple sclerosis. J Neuroimmunol. (2010) 221:73–80. doi: 10.1016/j.jneuroim.2010.02.005 20223525

[28] Hug A Korporal M Schröder I Haas J Glatz K Storch-Hagenlocher B. Thymic export function and T cell homeostasis in patients with relapsing remitting multiple sclerosis. J Immunol. (2003) 171:432–7. doi: 10.4049/jimmunol.171.1.432 12817027

[29] Haegert DG Hackenbroch JD Duszczyszyn D Fitz-Gerald L Zastepa E Mason H. Reduced thymic output and peripheral naive CD4 T-cell alterations in primary progressive multiple sclerosis (PPMS). J neuroimmunology. (2011) 233:233–9. doi: 10.1016/j.jneuroim.2010.12.007 21272945

[30] Balint B Haas J Schwarz A Jarius S Fürwentsches A Engelhardt K. T-cell homeostasis in pediatric multiple sclerosis: old cells in young patients. Neurology. (2013) 81:784–92. doi: 10.1212/WNL.0b013e3182a2ce0e 23911752

[31] Amoriello R Mariottini A Ballerini C. Immunosenescence and autoimmunity: exploiting the T-cell receptor repertoire to investigate the impact of aging on multiple sclerosis. Front Immunol. (2021) 12:799380. doi: 10.3389/fimmu.2021.799380 34925384 PMC8673061

[32] D'Andrea V Biancari F Cavallotti D Malinovsky L Di Matteo FM Modesti A. Thymectomy and multiple sclerosis: ultrastructural study of an experimental model. G Chir. (1999) 20:119–24. 10217872

[33] Papadopoulos D Magliozzi R Mitsikostas DD Gorgoulis VG Nicholas RS. Aging, cellular senescence, and progressive multiple sclerosis. Front Cell Neurosci. (2020) 14:178. doi: 10.3389/fncel.2020.00178 32694983 PMC7338849

[34] Thomas R Wang W Su D-M. Contributions of age-related thymic involution to immunosenescence and inflammaging. Immun Ageing. (2020) 17:2. doi: 10.1186/s12979-020-0173-8 31988649 PMC6971920

